# Diaqua­[*N*,*N*′-bis­(3-carboxy­prop-2-enoyl)pyridine-2,6-dicarbo­hydrazidato(2–)]cadmium(II) *N*,*N*-dimethyl­formamide disolvate

**DOI:** 10.1107/S1600536809011003

**Published:** 2009-03-31

**Authors:** Quanfu Cao, Dacheng Li

**Affiliations:** a College of Chemistry and Chemical Engineering, Liaocheng University, Liaocheng 252059, People’s Republic of China

## Abstract

In the title complex, [Cd(C_15_H_11_N_5_O_8_)(H_2_O)_2_]·2C_3_H_7_NO, the Cd^II^ ion is located on a twofold rotation axis and is seven-coordinated in a distorted penta­gonal-bipyramidal manner. The asymmetric unit comprises one metal ion, one doubly deprotonated *N*,*N*′-bis­(3-carboxy­prop-2-eno­yl)pyridine-2,6-dicarbohydrazide ligand, two coordinating water mol­ecules and two dimethyl­formamide solvent mol­ecules. In the crystal, a two-dimensional network is formed through N—H⋯O and O—H⋯O hydrogen bonds.

## Related literature

For polydimensional supermolecular architectures formed by aromatic hydrazides through hydrogen bonds and π–π inter­actions, see: Bacchi *et al.* (1993 ▶); Bermejo *et al.* (1999 ▶). The condensation products of 2,6-picolylhydrazide with anhydrides have been found to adopt a penta­gonal-bipyramidal coordination in various metal complexes, see: Pelizzi *et al.* (1987 ▶); Wang *et al.* (2005 ▶). For the chelating behaviour of *N*,*N*′-acetyl-2,6-picolylhydrazide with Fe^3+^, see: Cao *et al.* (2008 ▶). For our continuing study of aroylhydrazides, see: Dou *et al.* (2006 ▶). For Cd—O(carbon­yl) bond lengths in other seven-coordinated penta­gonal-bipyramidal cadmium complexes, see: Charles *et al.* (1983 ▶).
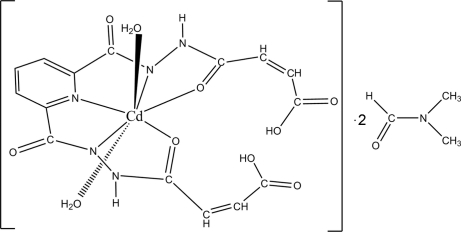


## Experimental

### 

#### Crystal data

[Cd(C_15_H_11_N_5_O_8_)(H_2_O)_2_]·2C_3_H_7_NO
*M* *_r_* = 683.91
Monoclinic, 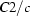

*a* = 18.6176 (2) Å
*b* = 12.6065 (8) Å
*c* = 12.0038 (6) Å
β = 99.51°
*V* = 2778.6 (2) Å^3^
*Z* = 4
Mo *K*α radiation
μ = 0.86 mm^−1^
*T* = 298 K
0.20 × 0.18 × 0.17 mm

#### Data collection

Siemens SMART CCD area-detector diffractometer
Absorption correction: multi-scan (*SADABS*; Sheldrick, 1996 ▶) *T*_min_ = 0.847, *T*_max_ = 0.868
6846 measured reflections
2448 independent reflections
2071 reflections with *I* > 2σ(*I*)
*R*_int_ = 0.028

#### Refinement

*R*[*F* ^2^ > 2σ(*F* ^2^)] = 0.027
*wR*(*F* ^2^) = 0.075
*S* = 1.00
2448 reflections
189 parameters
H-atom parameters constrained
Δρ_max_ = 0.73 e Å^−3^
Δρ_min_ = −0.48 e Å^−3^

### 

Data collection: *SMART* (Siemens, 1996 ▶); cell refinement: *SAINT* (Siemens, 1996 ▶); data reduction: *SAINT*; program(s) used to solve structure: *SHELXS97* (Sheldrick, 2008 ▶); program(s) used to refine structure: *SHELXL97* (Sheldrick, 2008 ▶); molecular graphics: *SHELXTL* (Sheldrick, 2008 ▶); software used to prepare material for publication: *SHELXTL*.

## Supplementary Material

Crystal structure: contains datablocks global, I. DOI: 10.1107/S1600536809011003/kp2207sup1.cif

Structure factors: contains datablocks I. DOI: 10.1107/S1600536809011003/kp2207Isup2.hkl

Additional supplementary materials:  crystallographic information; 3D view; checkCIF report

## Figures and Tables

**Table 1:** Selected bond lengths (Å)

Cd1—N2^i^	2.287 (2)
Cd1—O5^i^	2.3412 (19)
Cd1—N1	2.387 (3)
Cd1—O2^i^	2.4441 (19)
N2—N3	1.369 (3)

Symmetry code: (i) 
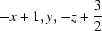
.

**Table 2:** Hydrogen-bond geometry (Å, °)

*D*—H⋯*A*	*D*—H	H⋯*A*	*D*⋯*A*	*D*—H⋯*A*
O5—H5*A*⋯O4^ii^	0.85	1.97	2.802 (3)	165
O5—H5*B*⋯O1^iii^	0.85	1.84	2.685 (3)	174
N3—H3*A*⋯O6	0.86	1.97	2.808 (3)	163
O3—H3⋯O2	0.82	1.68	2.498 (3)	175

Symmetry codes: (ii) 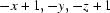; (iii) 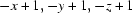.

**Fig. 1.**
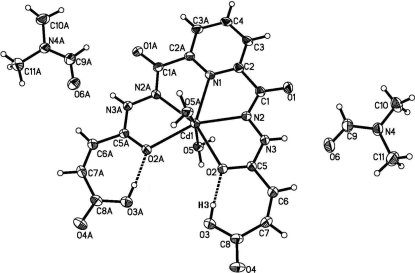
The molecular stucture of the complex (I) showing the atomic numbering scheme. Displacement ellipsoids are drawn at the 30% probability level. [Symmetry codes: -x + 1,y,-z + 3/2]

**Fig. 2.**
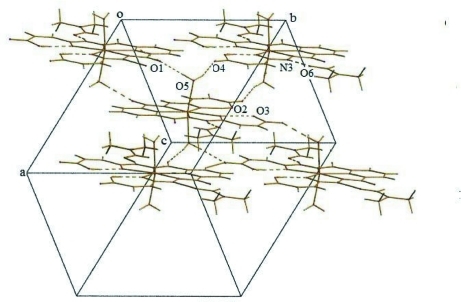
Part of the crystal structure of the complex, showing hydrogen bonds as dashed lines. [Symmetry codes: (i) -x + 1, -y, -z + 1; (ii) -x + 1, -y + 1, -z + 1].

## supplementary crystallographic information

### Comment

Containing N, O and other coordinating sites, aromatic hydrazides can form poly-dimensional supermolecular architectures through hydrogen bonds and *π*-π interactions (Bacchi *et al.*, 1993, Bermejo *et al.*, 1999). The condensation products of 2,6-picolylhydrazide with anhydrides have been found to adopt a pentagonal-bipyramidal stereochemistry in various metal complexes, in which they may participate as neutral and/or dianionic ligands (Pelizzi, *et al.*, 1987, Wang *et al.*, 2005). Previously we have examined the chelating behaviour of *N*,*N*'-acetyl-2,6-picolylhydrazide with Fe^3+^ (Cao, *et al.*, 2008). As a part of continuing study of our research on aroylhydrazide in our laboratory (Dou, *et al.*, 2006), we synthesized *N*,*N*'-bis(3-carboxy-*cis*-propenoyl)- 2,6-picolyldihydrazide and obtained its Cd(II) complex (I).

The molecular structure of the complex (Fig. 1) and its characteristic geometry parameteres (Table 1) reveal one cadmium ion which is located on the 2-fold rotation axis, one deprotonated ligand, two coordinated H_2_O molecules and two solvent DMF molecules. The divalent anionic H_2_*L*^2-^ acts as a pentadentate chelating ligand to two cadmium atoms.The remainder coordinating sites of Cd^2+^ are occupied by two O atoms from water molecules in *trans*-positions which complete the seven-coordinated pentagonal- bipyramid. Two deprotonated amide nitrogen atoms, two carbonyl O atoms, one pyridine N atom complete the equatorial plane and the mean deviation is 0.0064 Å indicating that the five atoms are ideally coplanar. Such planarity was observed in [Cd(H_2_daps)Cl_2_](CHCl_3_)(CH_3_OH) (less than 0.007 Å) (H_2_daps = 2,6-diacetylpridine bis(salicyloylhydrazone) (Pelizzi, *et al.*, 1987). The Cd—N distances are in the range of 2.287 (2) Å to 2.387 (3) Å; its average value of 2.320 (2) Å is  shorter than those observed in [Cd(*L*^'^)(1.5H_2_O)]n (*L*' = *N*,*N*'-bis(4-pyridylcarboxyl)- 2,6-pyridine dicarbohydrazide) (Wang *et al.*, 2005) and [Cd(H_2_daps)Cl_2_](CHCl_3_)(CH_3_OH) (Pelizzi, *et al.*, 1987). Both, two Cd—*O*(carbonyl) bond lengths (2.4441 (19) Å) are comparable to those in other seven-coordinated pentagonal-bipyramidal cadmium complexes (Charles *et al.*, 1983). The Cd—O (water) distance is 2.341 (2) Å, being shorter than the mean lengths of Cd—O in the the equatorial plane of 2.444 (19) Å.

The crystal structure of the title complex is predominantly determined by N—H···O and O—H···O hydrogen bonds (Table 2 and Fig. 2) generating 2-D network.

### Experimental

All chemicals were of reagent grade and were used without further purification. A solution of cadmium nitrate tetrahydrate (2 mmol, 0.457 g) dissolved in methanol (10 ml) was added dropwise to a DMF solution containing the ligand (2 mmol, 0.783 g). The mixture was stirred at room temperature for 6 h and then filtered.The filtrate was left to evaporate slowly at room temperature and yellow block-shaped crystals suitable for X-ray diffraction analysis were obtained after three weeks (m.p. >573 K). Elemental analysis calculated for (I): C: 36.88, H: 4.27, N: 14.34%; found: C: 36.11, H: 4.66, N: 14.02%. IR (KBr pellet, cm^-1^): 3467 (O—H), 3134 (N—H), 1709 (C=O) (acid carboxyl segment), 1647 (C=C).

### Refinement

All H atoms were placed geometrically and treated as riding on their parent atoms, with pyridine C—H distances of 0.930 Å, hydrazide N—H distances of 0.860 Å, alkene C–H distances of 0.930 Å, methyl C—H distances of 0.960 Å, and with *U*_iso_(H) = 1.2 *U*_eq_(C,*O*) and 1.5 *U*_eq_ for methyl and hydroxy groups.

### Crystal data

**Table:**

[Cd(C_15_H_11_N_5_O_8_)(H_2_O)_2_]·2C_3_H_7_NO	*F*(000) = 1392
*M**_r_* = 683.91	*D*_x_ = 1.635 Mg m^−^^3^
Monoclinic, *C*2/*c*	Mo *K*α radiation, λ = 0.71073 Å
Hall symbol: -C 2yc	Cell parameters from 3321 reflections
*a* = 18.6176 (2) Å	θ = 2.5–27.6°
*b* = 12.6065 (8) Å	µ = 0.86 mm^−^^1^
*c* = 12.0038 (6) Å	*T* = 298 K
β = 99.51°	Block, yellow
*V* = 2778.6 (2) Å^3^	0.20 × 0.18 × 0.17 mm
*Z* = 4	

### Data collection

**Table:**

Siemens SMART CCD area-detector diffractometer	2448 independent reflections
Radiation source: fine-focus sealed tube	2071 reflections with *I* > 2σ(*I*)
graphite	*R*_int_ = 0.028
φ and ω scans	θ_max_ = 25.0°, θ_min_ = 2.0°
Absorption correction: multi-scan (*SADABS*; Sheldrick, 1996)	*h* = −22→21
*T*_min_ = 0.847, *T*_max_ = 0.868	*k* = −14→8
6846 measured reflections	*l* = −14→14

### Refinement

**Table:**

Refinement on *F*^2^	Primary atom site location: structure-invariant direct methods
Least-squares matrix: full	Secondary atom site location: difference Fourier map
*R*[*F*^2^ > 2σ(*F*^2^)] = 0.027	Hydrogen site location: inferred from neighbouring sites
*wR*(*F*^2^) = 0.075	H-atom parameters constrained
*S* = 1.00	*w* = 1/[σ^2^(*F*_o_^2^) + (0.0426*P*)^2^ + 1.9365*P*] where *P* = (*F*_o_^2^ + 2*F*_c_^2^)/3
2448 reflections	(Δ/σ)_max_ = 0.001
189 parameters	Δρ_max_ = 0.73 e Å^−^^3^
0 restraints	Δρ_min_ = −0.48 e Å^−^^3^

### Special details

**Table:**

Geometry. All e.s.d.'s (except the e.s.d. in the dihedral angle between two l.s. planes) are estimated using the full covariance matrix. The cell e.s.d.'s are taken into account individually in the estimation of e.s.d.'s in distances, angles and torsion angles; correlations between e.s.d.'s in cell parameters are only used when they are defined by crystal symmetry. An approximate (isotropic) treatment of cell e.s.d.'s is used for estimating e.s.d.'s involving l.s. planes.

### Fractional atomic coordinates and isotropic or equivalent isotropic displacement parameters (Å^2^)

**Table:**

	*x*	*y*	*z*	*U*_iso_*/*U*_eq_	
Cd1	0.5000	0.32546 (2)	0.7500	0.03647 (12)	
N1	0.5000	0.5148 (2)	0.7500	0.0315 (7)	
N2	0.56574 (12)	0.39595 (18)	0.62292 (19)	0.0356 (5)	
N3	0.59654 (12)	0.32818 (17)	0.55520 (19)	0.0346 (5)	
H3A	0.6204	0.3519	0.5049	0.042*	
N4	0.73608 (14)	0.4660 (2)	0.2710 (2)	0.0474 (6)	
O1	0.60699 (11)	0.53782 (16)	0.53204 (17)	0.0457 (5)	
O2	0.55458 (11)	0.18980 (15)	0.64485 (17)	0.0413 (5)	
O3	0.55912 (13)	−0.00807 (17)	0.6368 (2)	0.0596 (6)	
H3	0.5574	0.0566	0.6435	0.089*	
O4	0.60364 (16)	−0.12393 (19)	0.5313 (2)	0.0733 (8)	
O5	0.39670 (11)	0.29907 (15)	0.61261 (16)	0.0420 (5)	
H5A	0.3988	0.2396	0.5800	0.050*	
H5B	0.3967	0.3475	0.5634	0.050*	
O6	0.68682 (14)	0.3634 (2)	0.3932 (2)	0.0656 (7)	
C1	0.57294 (15)	0.4982 (2)	0.6033 (2)	0.0345 (6)	
C2	0.53537 (14)	0.5671 (2)	0.6791 (2)	0.0340 (6)	
C3	0.53679 (16)	0.6767 (2)	0.6772 (3)	0.0403 (7)	
H3B	0.5619	0.7127	0.6280	0.048*	
C4	0.5000	0.7315 (3)	0.7500	0.0414 (10)	
H4	0.5000	0.8053	0.7500	0.050*	
C5	0.58854 (14)	0.2254 (2)	0.5694 (2)	0.0336 (6)	
C6	0.62026 (18)	0.1570 (2)	0.4921 (3)	0.0460 (8)	
H6	0.6438	0.1921	0.4403	0.055*	
C7	0.62029 (19)	0.0514 (2)	0.4854 (3)	0.0523 (8)	
H7	0.6419	0.0254	0.4264	0.063*	
C8	0.59292 (18)	−0.0317 (2)	0.5527 (3)	0.0483 (8)	
C9	0.70087 (17)	0.4503 (3)	0.3561 (3)	0.0519 (8)	
H9	0.6854	0.5100	0.3910	0.062*	
C10	0.75008 (19)	0.5723 (3)	0.2334 (3)	0.0612 (10)	
H10A	0.7260	0.6230	0.2741	0.092*	
H10B	0.7320	0.5786	0.1540	0.092*	
H10C	0.8016	0.5856	0.2472	0.092*	
C11	0.7598 (2)	0.3769 (3)	0.2101 (3)	0.0672 (10)	
H11A	0.7465	0.3119	0.2431	0.101*	
H11B	0.8117	0.3796	0.2143	0.101*	
H11C	0.7368	0.3801	0.1325	0.101*	

### Atomic displacement parameters (Å^2^)

**Table:**

	*U*^11^	*U*^22^	*U*^33^	*U*^12^	*U*^13^	*U*^23^
Cd1	0.0481 (2)	0.02519 (17)	0.03978 (19)	0.000	0.01812 (13)	0.000
N1	0.0379 (17)	0.0217 (16)	0.0355 (18)	0.000	0.0083 (14)	0.000
N2	0.0455 (13)	0.0251 (12)	0.0391 (14)	0.0013 (10)	0.0155 (11)	−0.0008 (10)
N3	0.0424 (13)	0.0293 (13)	0.0351 (13)	−0.0007 (10)	0.0150 (10)	0.0013 (10)
N4	0.0528 (15)	0.0427 (15)	0.0515 (16)	0.0070 (12)	0.0226 (13)	0.0104 (12)
O1	0.0627 (13)	0.0346 (12)	0.0450 (12)	0.0011 (10)	0.0240 (10)	0.0079 (9)
O2	0.0546 (12)	0.0284 (11)	0.0466 (12)	0.0010 (9)	0.0247 (10)	−0.0009 (9)
O3	0.0866 (17)	0.0307 (12)	0.0707 (16)	−0.0033 (11)	0.0394 (14)	−0.0011 (11)
O4	0.116 (2)	0.0311 (14)	0.0787 (19)	−0.0008 (13)	0.0324 (16)	−0.0118 (12)
O5	0.0580 (12)	0.0299 (10)	0.0391 (11)	−0.0008 (9)	0.0109 (9)	0.0010 (9)
O6	0.0778 (17)	0.0552 (15)	0.0728 (17)	−0.0020 (13)	0.0393 (14)	0.0140 (13)
C1	0.0398 (15)	0.0314 (16)	0.0321 (15)	−0.0003 (12)	0.0055 (12)	0.0047 (12)
C2	0.0387 (15)	0.0285 (15)	0.0346 (15)	0.0005 (12)	0.0053 (12)	0.0035 (12)
C3	0.0521 (17)	0.0254 (15)	0.0437 (17)	−0.0033 (13)	0.0093 (14)	0.0056 (12)
C4	0.058 (3)	0.020 (2)	0.047 (3)	0.000	0.008 (2)	0.000
C5	0.0384 (15)	0.0285 (16)	0.0358 (16)	0.0001 (12)	0.0119 (12)	0.0030 (12)
C6	0.0576 (19)	0.0354 (18)	0.0506 (19)	0.0015 (14)	0.0253 (15)	0.0005 (14)
C7	0.069 (2)	0.0397 (19)	0.054 (2)	0.0049 (16)	0.0299 (17)	−0.0071 (15)
C8	0.064 (2)	0.0311 (18)	0.0510 (19)	−0.0008 (14)	0.0121 (16)	−0.0051 (14)
C9	0.0539 (19)	0.050 (2)	0.056 (2)	0.0015 (16)	0.0210 (16)	0.0046 (16)
C10	0.062 (2)	0.054 (2)	0.072 (3)	0.0002 (17)	0.0235 (19)	0.0211 (19)
C11	0.079 (3)	0.060 (2)	0.069 (3)	0.012 (2)	0.032 (2)	0.004 (2)

### Geometric parameters (Å, °)

**Table:**

Cd1—N2^i^	2.287 (2)	O5—H5A	0.8500
Cd1—N2	2.287 (2)	O5—H5B	0.8500
Cd1—O5^i^	2.3412 (19)	O6—C9	1.227 (4)
Cd1—O5	2.3412 (19)	C1—C2	1.511 (4)
Cd1—N1	2.387 (3)	C2—C3	1.382 (4)
Cd1—O2^i^	2.4441 (19)	C3—C4	1.382 (4)
Cd1—O2	2.4441 (19)	C3—H3B	0.9300
N1—C2^i^	1.334 (3)	C4—C3^i^	1.382 (4)
N1—C2	1.334 (3)	C4—H4	0.9300
N2—C1	1.321 (4)	C5—C6	1.460 (4)
N2—N3	1.369 (3)	C6—C7	1.335 (4)
N3—C5	1.319 (3)	C6—H6	0.9300
N3—H3A	0.8600	C7—C8	1.465 (4)
N4—C9	1.316 (4)	C7—H7	0.9300
N4—C11	1.448 (4)	C9—H9	0.9300
N4—C10	1.452 (4)	C10—H10A	0.9600
O1—C1	1.250 (3)	C10—H10B	0.9600
O2—C5	1.269 (3)	C10—H10C	0.9600
O3—C8	1.309 (4)	C11—H11A	0.9600
O3—H3	0.8200	C11—H11B	0.9600
O4—C8	1.214 (4)	C11—H11C	0.9600
			
N2^i^—Cd1—N2	134.27 (11)	O1—C1—C2	121.3 (2)
N2^i^—Cd1—O5^i^	93.05 (8)	N2—C1—C2	112.5 (2)
N2—Cd1—O5^i^	93.28 (8)	N1—C2—C3	121.2 (3)
N2^i^—Cd1—O5	93.28 (8)	N1—C2—C1	115.2 (2)
N2—Cd1—O5	93.05 (8)	C3—C2—C1	123.6 (3)
O5^i^—Cd1—O5	163.66 (9)	C4—C3—C2	118.5 (3)
N2^i^—Cd1—N1	67.13 (6)	C4—C3—H3B	120.8
N2—Cd1—N1	67.13 (6)	C2—C3—H3B	120.8
O5^i^—Cd1—N1	98.17 (5)	C3^i^—C4—C3	120.0 (4)
O5—Cd1—N1	98.17 (5)	C3^i^—C4—H4	120.0
N2^i^—Cd1—O2^i^	67.27 (7)	C3—C4—H4	120.0
N2—Cd1—O2^i^	158.46 (8)	O2—C5—N3	121.3 (2)
O5^i^—Cd1—O2^i^	84.26 (7)	O2—C5—C6	123.1 (2)
O5—Cd1—O2^i^	84.33 (7)	N3—C5—C6	115.6 (2)
N1—Cd1—O2^i^	134.40 (4)	C7—C6—C5	129.2 (3)
N2^i^—Cd1—O2	158.46 (8)	C7—C6—H6	115.4
N2—Cd1—O2	67.27 (7)	C5—C6—H6	115.4
O5^i^—Cd1—O2	84.33 (7)	C6—C7—C8	132.6 (3)
O5—Cd1—O2	84.26 (7)	C6—C7—H7	113.7
N1—Cd1—O2	134.40 (4)	C8—C7—H7	113.7
O2^i^—Cd1—O2	91.20 (9)	O4—C8—O3	119.9 (3)
C2^i^—N1—C2	120.7 (3)	O4—C8—C7	118.9 (3)
C2^i^—N1—Cd1	119.64 (16)	O3—C8—C7	121.2 (3)
C2—N1—Cd1	119.64 (16)	O6—C9—N4	125.4 (3)
C1—N2—N3	116.0 (2)	O6—C9—H9	117.3
C1—N2—Cd1	125.41 (19)	N4—C9—H9	117.3
N3—N2—Cd1	118.45 (16)	N4—C10—H10A	109.5
C5—N3—N2	118.0 (2)	N4—C10—H10B	109.5
C5—N3—H3A	121.0	H10A—C10—H10B	109.5
N2—N3—H3A	121.0	N4—C10—H10C	109.5
C9—N4—C11	120.5 (3)	H10A—C10—H10C	109.5
C9—N4—C10	121.2 (3)	H10B—C10—H10C	109.5
C11—N4—C10	118.3 (3)	N4—C11—H11A	109.5
C5—O2—Cd1	114.84 (16)	N4—C11—H11B	109.5
C8—O3—H3	109.5	H11A—C11—H11B	109.5
Cd1—O5—H5A	110.8	N4—C11—H11C	109.5
Cd1—O5—H5B	107.1	H11A—C11—H11C	109.5
H5A—O5—H5B	108.0	H11B—C11—H11C	109.5
O1—C1—N2	126.2 (3)		
			
N2^i^—Cd1—N1—C2^i^	0.87 (14)	O5—Cd1—O2—C5	92.38 (19)
N2—Cd1—N1—C2^i^	−179.13 (14)	N1—Cd1—O2—C5	−3.4 (2)
O5^i^—Cd1—N1—C2^i^	−89.00 (14)	O2^i^—Cd1—O2—C5	176.6 (2)
O5—Cd1—N1—C2^i^	91.00 (14)	N3—N2—C1—O1	−2.6 (4)
O2^i^—Cd1—N1—C2^i^	0.95 (15)	Cd1—N2—C1—O1	−177.9 (2)
O2—Cd1—N1—C2^i^	−179.05 (15)	N3—N2—C1—C2	178.2 (2)
N2^i^—Cd1—N1—C2	−179.13 (14)	Cd1—N2—C1—C2	2.8 (3)
N2—Cd1—N1—C2	0.87 (14)	C2^i^—N1—C2—C3	0.3 (2)
O5^i^—Cd1—N1—C2	91.00 (14)	Cd1—N1—C2—C3	−179.7 (2)
O5—Cd1—N1—C2	−89.00 (14)	C2^i^—N1—C2—C1	−179.9 (2)
O2^i^—Cd1—N1—C2	−179.05 (15)	Cd1—N1—C2—C1	0.1 (2)
O2—Cd1—N1—C2	0.95 (15)	O1—C1—C2—N1	179.0 (2)
N2^i^—Cd1—N2—C1	−2.1 (2)	N2—C1—C2—N1	−1.7 (3)
O5^i^—Cd1—N2—C1	−99.6 (2)	O1—C1—C2—C3	−1.2 (4)
O5—Cd1—N2—C1	95.5 (2)	N2—C1—C2—C3	178.1 (3)
N1—Cd1—N2—C1	−2.1 (2)	N1—C2—C3—C4	−0.6 (4)
O2^i^—Cd1—N2—C1	177.7 (2)	C1—C2—C3—C4	179.6 (2)
O2—Cd1—N2—C1	178.0 (3)	C2—C3—C4—C3^i^	0.29 (19)
N2^i^—Cd1—N2—N3	−177.3 (2)	Cd1—O2—C5—N3	3.8 (3)
O5^i^—Cd1—N2—N3	85.19 (19)	Cd1—O2—C5—C6	−175.4 (2)
O5—Cd1—N2—N3	−79.74 (19)	N2—N3—C5—O2	−1.3 (4)
N1—Cd1—N2—N3	−177.3 (2)	N2—N3—C5—C6	177.9 (2)
O2^i^—Cd1—N2—N3	2.5 (3)	O2—C5—C6—C7	−0.1 (5)
O2—Cd1—N2—N3	2.74 (17)	N3—C5—C6—C7	−179.3 (4)
C1—N2—N3—C5	−177.8 (2)	C5—C6—C7—C8	−3.2 (7)
Cd1—N2—N3—C5	−2.1 (3)	C6—C7—C8—O4	−176.1 (4)
N2^i^—Cd1—O2—C5	176.8 (2)	C6—C7—C8—O3	2.0 (6)
N2—Cd1—O2—C5	−3.36 (18)	C11—N4—C9—O6	2.1 (5)
O5^i^—Cd1—O2—C5	−99.33 (19)	C10—N4—C9—O6	−180.0 (3)

Symmetry codes: (i) −*x*+1, *y*, −*z*+3/2.

### Hydrogen-bond geometry (Å, °)

**Table:**

*D*—H···*A*	*D*—H	H···*A*	*D*···*A*	*D*—H···*A*
O5—H5A···O4^ii^	0.85	1.97	2.802 (3)	165
O5—H5B···O1^iii^	0.85	1.84	2.685 (3)	174
N3—H3A···O6	0.86	1.97	2.808 (3)	163
O3—H3···O2	0.82	1.68	2.498 (3)	175

Symmetry codes: (ii) −*x*+1, −*y*, −*z*+1; (iii) −*x*+1, −*y*+1, −*z*+1.
